# Supplementary material for: Stationary correlation pattern in highly non-stationary MEG recordings of healthy subjects and its relation to former EEG studies
Source: PLoS One. 2024 Oct 22;19(10):e0307378. doi: 10.1371/journal.pone.0307378 (PMC11495582; doi:10.1371/journal.pone.0307378)
Supplement: Attachment — Submitted filename: Rebuttal_Letter.pdf [file pone.0307378.s002.pdf]

The reviewer claims that: "The authors have addressed most of my comments. I still thing the Abstract is no of high quality. Authors did not address my comment on the abstract and still using words like strikingly similar without quantities although in their response they write that they have done that. They did not address my comment on Fig. 1. The figure added to illustrate the experimental design is not clear. There are some careless English mistakes like "hypothesise". Authors need to improve the English language."

We changed Figure 1 and Figure 2, hoping that the new versions of both figures are now satisfying. However, we would like to remark that it is certainly no sufficient to state that a Figure is not clear, but one should specify possible deficiencies. Furthermore, in contrast to the statement of the reviewer we added labels to Fig. 1 and explained the algorithm in the main text. However, here we propose a new design of Figure 1.

We changed the abstract, actually we rewrote it completely. However, we think that using a term like "strikingly" in this part of a manuscript is by no means a deficient redaction and we cannot see any rational behind the claim of the reviewer. We are still of the opinion that the use of this term was justified, especially since we quantitatively demonstrate in the main text how highly (strikingly) similar the correlation structures are.

Finally, using the term "hypothesise" or "hypothesize" is a question of using British or the American way of English writing. But it is certainly not a mistake, as the reviewer claims. Besides, we recommend the reviewer being more careful in the redaction of his report (see e.g. "I still thing the Abstract is no of high quality"), especially when he/she criticizes the English of others. Being consistent within the whole text we did not change this term.
